# Differential pathways linking social interaction and Loneliness among university students in the age of artificial intelligence: the mediating role of Social Capital

**DOI:** 10.3389/fpsyg.2026.1884079

**Published:** 2026-09-01

**Authors:** Juqin Yin, Hengkui Zhuo, Siyan Zhou, Zhinian Li

**Affiliations:** 1 School of Economics/Hangzhou Dianzi University, Hangzhou, China; 2 School of Management, Zhejiang University, Hangzhou, China

**Keywords:** artificial intelligence, Loneliness, Social Capital, social interaction, university students

## Abstract

Based on Social Capital theory, this study explores the mechanisms linking emerging forms of social interaction and individual Loneliness among university students in the age of artificial intelligence (AI), from the perspective of Social Capital accumulation within AI-mediated social environments. Using survey data collected from university students, a Partial Least Squares Structural Equation Modeling (PLS-SEM) approach was employed to analyze the relationships among key variables. Specifically, social interaction preferences (including behavioral preferences and image presentation preferences) and embodied social competence were treated as independent variables. Social Capital was modeled as a higher-order latent construct comprising Bonding, Bridging, and Linking Social Capital, which jointly mediated the associations between social interaction characteristics and Loneliness.; and Loneliness was specified as the dependent variable. The results reveal differentiated mediating mechanisms within the AI-driven social interaction system. Online social interaction preference exhibits a dual-path effect on Loneliness: it directly increases Loneliness, while also exerting an indirect mitigating effect through Social Capital; however, this indirect effect is notably weak, suggesting limited psychological compensation. In contrast, embodied social competence emerges as the strongest predictor of Social Capital and significantly reduces Loneliness through higher levels of Social Capital. Furthermore, different sources of Social Capital demonstrate distinct effects in the context of AI-mediated social environments. The pathway linking online social interaction preferences to Social Capital was comparatively weaker, whereas the pathway from Offline Social Competence to Social Capital was substantially stronger. These findings suggest that different pathways of Social Capital accumulation may be associated with varying levels of protection against Loneliness. Overall, this study identifies differentiated associations among social interaction characteristics, Social Capital, and Loneliness, extends the application of Social Capital theory in AI-enabled socio-technical contexts, and provides important implications for promoting university students' social development and mental wellbeing.

## 1 Introduction

Artificial intelligence (AI) technologies are profoundly reshaping the social landscape of university students. Online platforms, intelligent interactive tools, and algorithm-driven information environments have greatly expanded opportunities for connection, creatively enlarging the scope of social engagement and promoting greater flexibility and autonomy in interaction. At the same time, these developments have given rise to new forms of sociality characterized by human–machine collaboration and the integration of the virtual and the real. However, alongside this expansion of connectivity, levels of Loneliness among university students have shown a persistent upward trend. Uncovering the underlying mechanisms linking contemporary forms of social interaction and Loneliness, and explaining the paradox of “more interaction yet deeper Loneliness,” has become a important issue in youth studies.

A review of existing research on AI-mediated social use and Loneliness among university students reveals a paradoxical pattern in which increased digital social engagement is associated with heightened Loneliness. Existing research has reached a basic consensus on the macro-level explanations of social compensation and substitution effects; they remain limited by the assumption of homogeneity in Social Capital, and thus fail to adequately explain the differential effects of online and offline social pathways.

To address this gap, the present study develops a conceptual model examining the associations among social interaction characteristics, Social Capital, and Loneliness grounded in a multidimensional framework of Social Capital. Focusing on university students as a population at a critical stage of social development, this study employs Partial Least Squares Structural Equation Modeling (PLS-SEM) to examine whether online social interaction preferences and embodied social competence are differentially associated with Loneliness through Social Capital. In doing so, the study offers a novel explanation for the “connectivity paradox” in the age of artificial intelligence and provides practical implications for promoting university students' social development and mental wellbeing. The remainder of this paper proceeds as follows: it reviews the relevant literature, develops research hypotheses, describes the sample and methodology, and presents the empirical analysis.

## 2 Literature review

### 2.1 Phenomenon observation: the paradox of loneliness amid the proliferation of digital social interactions

The rapid development of artificial intelligence (AI) has fundamentally transformed so-cial interaction patterns among university students, contributing to increasingly diversi-fied and lightweight forms of communication. Generative and emotionally supportive AI systems, such as AI chatbots and intelligent companions, provide young adults with immediate, low-pressure, and personalized interaction experiences (Krakowski, 2025; Kim M. et al., 2025; Zhang et al., 2025). Emerging so-cial practices characterized by weak-tie relationships, such as “companion-based social-izing” and low-commitment interaction patterns, have broadened opportunities for social engagement and increased communication efficiency. However, these developments may simultaneously contribute to more superficial interpersonal relationships and weakened emotional attachment (Zhang and Che, 2026; Duan, 2025; Chen, 2026; Guo, 2025; Dong et al., 2025).

The innovation in social interaction methods has not correspondingly improved mental health. Extensive research has revealed a “Loneliness paradox”: while artificial intelligence has expanded social reach and interaction frequency (Zhang et al., 2025), feelings of Loneliness and social anxiety have become increasingly prevalent among college students (Tan, 2025; Nowland et al., 2018; Ganie et al., 2026). Local survey data provides direct evidence: 75.2% of surveyed young people use social media fre-quently, yet 64.3% admit that “deep-seated Loneliness remains difficult to alleviate”; the adoption rate of generative AI among young people has reached 51.8%, with 16.5% of us-ers viewing AI as an “intimate other” for emotional solace (Yin and Gao, 2026).

Regarding usage patterns, passive and reality-avoidant AI usage tends to exacerbate lone-liness (Herbener and Damholdt, 2025), with domestic studies confirming a significant correlation between such usage motivations and higher levels of Loneliness (Hou et al., 2025). Longitudinal evidence further indicates that AI interaction frequency influences prosocial behavior and problematic phone use through feelings of Loneliness (Sun et al., 2025). In terms of user characteristics, adoles-cents who prefer relational AI dialogue styles often exhibit poorer family and peer rela-tionship quality, as well as higher levels of stress and anxiety, highlighting that socially vulnerable individuals are more susceptible to being drawn to AI's emotional functions (Kim P. et al., 2025). Regarding usage consequences, greater satisfaction and positive experiences with AI chatbots are associated with heightened concerns about the loss of interpersonal connec-tions and emotional support (Al-Zahrani, 2025); excessive reliance on AI for emotional compensation weakens offline social motivation, leading to an imbalance characterized by “online activ-ity but offline detachment” (Yang et al., 2025; Duong et al., 2025). A randomized controlled trial found that a culturally adapted, CBT-based AI chatbot effectively reduced Loneliness among Chinese university students, with stronger effects for those under high financial stress (Wang et al., 2025). Even in instrumental usage contexts, frequent ChatGPT use shows a positive correlation with Loneliness and a negative correlation with social support (Zhao et al., 2025).

Within this context, social competence has become an important factor shaping both AI use and psychological outcomes. Existing research suggests that AI exerts a dual effect on university students' social development. On the one hand, intelligent technologies may improve access to social support and facilitate communication; on the other hand, exces-sive reliance on virtual interaction may weaken real-world social skills (Plechatá et al., 2025). Individuals with stronger social competence are more likely to use AI as a complementary resource, whereas those with weaker social skills may be more vulnerable to social anxiety, lone-liness, and avoidance of face-to-face interaction.

The aforementioned evidence indicates that focusing solely on the “quantity” of social in-teractions can no longer account for their complex effects. The coexistence of digital social prosperity and the proliferation of Loneliness points to a deeper theoretical question: how different modes of social interaction translate into social resources of varying quality, thereby exerting markedly distinct impacts on mental health.

### 2.2 Theoretical debate: dual explanations of social compensation and social replacement

Existing research generally recognizes the dual effects of AI-mediated social interaction on university students' social experiences and psychological wellbeing. The mechanisms underlying these effects can be interpreted through broader theoretical perspectives, in-cluding social learning theory and theories of technological alienation (Jia, 2023; Liu and Du, 2025; Chen et al., 2025; Feng and Li, 2026; Jiang et al., 2025; Sun, 2025). Two competing perspectives in particular have been widely used to explain the para-doxical effects of digital interaction.

The social compensation perspective argues that online environments can provide emo-tional support and interaction opportunities for individuals experiencing difficulties in real-world social settings, thereby reducing Loneliness and improving wellbeing (Zhang, 2025; Deng et al., 2026). Empirical studies indicate that emotionally supportive AI systems may enhance percep-tions of social support and improve interaction experiences by simulating emotional communication (Folk and Dunn, 2026; Liao et al., 2026).

In contrast, the social displacement perspective suggests that increased engagement in vir-tual environments may replace time and psychological resources previously devoted to of-fline interaction. Direct empirical support shows that when university students substitute human support with AI chatbots—for example, turning to AI rather than a librarian, professor, or student advisor—their sense of belonging and psychological wellbeing are negatively impacted, with Loneliness mediating this relationship (Crawford et al., 2024). Excessive reliance on technology may weaken the quality of interperson-al relationships and reduce the availability of real-world support networks, thereby con-tributing to Loneliness and social isolation (Hunt et al., 2018; Plechatá et al., 2025; Jia, 2023; Zhang, 2026; Wang and Liu, 2025; Jing et al., 2025). Overdependence on AI tools such as ChatGPT may further strengthen emotional reliance and social avoidance behav-iors, creating a cycle of “online dependence and offline detachment” (Ma, 2025; Yu and Yan, 2026; Wang and Yu, 2025).

### 2.3 Analytical framework: the introduction and findings of Social Capital theory

Social Capital has been widely recognized as an important mechanism linking social inter-action and psychological wellbeing. Within social interaction research, Social Capital is commonly conceptualized as resources embedded within social relationships and net-works. Building on classical Social Capital theories, Woolcock proposed a multidimen-sional framework consisting of bonding, bridging, and linking Social Capital (Putnam, 2000; Szreter and Woolcock, 2004). This framework provides an important analytical lens for understanding how different forms of social interaction shape psychological outcomes in AI-mediated environments.

A growing body of research indicates that Social Capital functions as a protective factor against Loneliness. Studies have shown that intimate interaction and self-disclosure through social media can reduce Loneliness by strengthening bonding and bridging Social Capital (Qi et al., 2025). Domestic studies similarly suggest that social media use influences Loneliness and depressive symptoms through Social Capital pathways. In addition, previous research has identified social competence as an important prerequisite for Social Capital accumulation (Jeong and Bae, 2025; Sohn et al., 2019; Bai et al., 2025; Li, 2024).

Recent studies have increasingly shifted attention from the quantity of Social Capital to-ward its quality and sources. Early research suggested that online and offline Social Capital differ qualitatively in terms of psychological support functions (Williams, 2007). More recent evidence indicates that face-to-face Social Capital demonstrates stronger positive associations with psychological wellbeing, whereas online Social Capital appears to have comparatively lim-ited effects on Loneliness reduction (Ye and Ho, 2024). Similarly, experimental findings suggest that in-teractions with highly supportive AI chatbots remain less effective than interactions with real human partners in alleviating Loneliness (Li et al., 2026).

### 2.4 Summary of the literature review: constructing a differential mediation model

In summary, the present review systematically examined prior studies on AI-mediated social interaction and Loneliness among university students, highlighting the paradox of “ increased connectedness yet heightened Loneliness” in the context of digital social expansion. Existing literature has largely reached a broad consensus regarding the macro-level explanatory perspectives of the social compensation effect and the social displacement effect. Nevertheless, several critical gaps remain.

First, there is a lack of integrative mechanism analysis, as prior research has seldom systematically examined the full pathway linking emerging social interaction patterns, Social Capital, and Loneliness. Second, existing studies often rely on a homogeneity assumption of Social Capital, overlooking qualitative differences in the sources and structures of Social Capital, thereby limiting their ability to explain the coexistence of “social prosperity” and the spread of Loneliness. Third, localized empirical evidence remains insufficient, particularly with respect to integrative model testing among Chinese university students.

To address these limitations, the present study adopts Michael Woolcock's Social Capital typology as the theoretical framework to examine how social interaction patterns in the digital era influence Loneliness among university students. Specifically, this study proposes a mediation model that investigates whether online social preference characteristics and offline embodied social competence are associated with Loneliness through Social Capital accumulation.

Building on this framework, the following sections further present the research hypotheses, sample design, and analytical methods employed for empirical testing.

## 3 Research design

### 3.1 Participants and procedure

This study employed a cross-sectional survey design to examine the relationships among social interaction characteristics, Social Capital, and Loneliness among university students in AI-mediated social environments.

Data were collected through an anonymous online questionnaire administered to students from multiple higher education institutions across Zhejiang Province, China. To enhance sample diversity and representativeness, the survey was distributed through university communication channels, student organizations, and academic networks, enabling participation from students with different academic backgrounds and disciplines. Participation was voluntary, and no financial incentives were provided.

Prior to completing the questionnaire, all participants were informed of the purpose of the study, the anonymous nature of participation, and their right to withdraw at any time without penalty. Electronic informed consent was obtained from all participants before participation. The study protocol was reviewed and approved by the Ethics Committee of Zhejiang University (Approval No. [2025] 009), and all procedures were conducted in accordance with relevant ethical guidelines and regulations.

A total of 835 questionnaires were returned. Data screening procedures were conducted before analysis, and questionnaires containing substantial missing data, obvious response irregularities, or patterns indicative of inattentive responding were excluded. After data cleaning, five questionnaires were removed, resulting in a final sample of 830 valid responses and a data-retention rate of 99.40%. Among the valid respondents, 50.84% were female and 49.16% were male. In terms of academic background, 64.10% were enrolled in humanities and social sciences disciplines, whereas 35.90% were enrolled in science and engineering disciplines.

### 3.2 Survey tools

#### 3.2.1 Measures

All latent constructs were measured using established scales adapted to the context of university students' social interaction in AI-mediated environments. Unless otherwise specified, items were assessed using a five-point Likert scale ranging from 1 (“not at all applicable”) to 5 (“very applicable”), with higher scores indicating higher levels of the corresponding construct. The Loneliness scale employed a four-point response format ranging from 1 (“never”) to 4 (“often”), with higher scores indicating greater perceived Loneliness.

The specific variable definitions are as follows:

- **Social interaction preference**: the degree of preference for and dependence on online social interaction. Higher scores indicate a stronger tendency to rely on social networking platforms for social engagement. Social Interaction Preference was measured using five items adapted from the Social Media Preference Scale (SMPS) and the AI Social Acceptance Scale. Example items included: “I prefer online social interaction to face-to-face interaction” and “When experiencing emotional difficulties, I tend to seek support through online communication (including AI chatbots) rather than face-to-face conversations.”
- **Social Presentation Preference**: the tendency to manage and construct one's image across online and offline contexts. Higher scores reflect a stronger inclination toward curating a favorable self-presentation on social media. Social Presentation Preference was measured using two items developed based on Goffman's dramaturgical theory and adapted from the Social Media Self-Presentation Focus Scale (SPAUSCIS). The scale assessed participants' tendency to manage and construct a favorable online self-image. Example items included: “I present a more ideal version of myself online” and “Maintaining a positive online image is important to me.”
- **Offline Social Competence**: individuals' social skills and interpersonal abilities. Higher scores indicate stronger embodied social competence in face-to-face interactions. Offline Social Competence was measured using eight items adapted from the Interpersonal Communication Competence Scale (ICCS). The scale covered four dimensions: verbal expression, emotional perception, nonverbal cue interpretation, and social adaptability. Example items included: “I can clearly express my thoughts and opinions to others,” “I can accurately perceive others' emotional changes,” and “I can quickly adapt to new face-to-face social situations.”
- **Social Capital**: the resources and support accessible through social networks. Higher scores indicate greater accumulation of Social Capital. Social Capital was conceptualized as a higher-order construct consisting of Bonding Social Capital, Bridging Social Capital, and Linking Social Capital, following Woolcock's Social Capital framework. Bonding Social Capital was measured using five items assessing trust, emotional support, reciprocity, and belonging within close interpersonal networks. Bridging Social Capital was measured using five items assessing access to diverse social networks, cross-group interaction, information exchange, and opportunities derived from heterogeneous relationships. Linking Social Capital was measured using five items assessing connections with formal organizations and institutions, trust in authority structures, and access to institutional resources and support.
- **Loneliness**: the subjective experience of Loneliness. Higher scores indicate higher levels of perceived Loneliness. Loneliness was measured using the shortened UCLA Loneliness Scale. Eight items assessed participants' subjective feelings of social isolation, emotional disconnection, and lack of meaningful interpersonal relationships. Example items included: “I feel isolated from others,” “I feel that no one truly understands me,” and “I often feel lonely even when surrounded by other people.”

#### 3.2.2 Scale adaptation and validation

The measurement instruments were adapted from established scales and relevant theoretical frameworks. For scales originally developed in English, a translation–back translation procedure was employed to ensure semantic equivalence, cultural appropriateness, and clarity of expression.

To establish content validity, the preliminary questionnaire was reviewed by five experts from the fields of psychology, communication studies, education, and sociology. The experts evaluated item relevance, construct correspondence, wording clarity, and suitability for university students. Based on their feedback, several items were revised or simplified.

Prior to the formal survey, a pilot study involving 20 university students was conducted to assess questionnaire comprehensibility and response burden. Participants reported that the questionnaire was easy to understand and required approximately 3–5 min to complete. Minor wording revisions were subsequently implemented before the formal administration.

Following data collection, measurement model evaluation indicated satisfactory reliability and validity. All constructs achieved acceptable levels of Cronbach's alpha, Composite Reliability (CR), and Average Variance Extracted (AVE). Standardized factor loadings exceeded the recommended threshold of 0.70, and additional assessments using HTMT, the Fornell–Larcker criterion, and cross-loading analyses supported adequate discriminant validity. Detailed results are presented in Table 1.

**Table 1:** Reliability and validity assessment of the measurement model.

Construct	Source	Items retained	Example item	Response format
Social interaction preference	Adapted from the Social Media Preference Scale (SMPS) and the AI Social Acceptance Scale	5	“I prefer online social interaction to face-to-face interaction.”	1–5
Social Presentation Preference	Developed based on Goffman's dramaturgical theory and adapted from the Social Media Self-Presentation Focus Scale (SPAUSCIS/LifeOnSoMe)	2	“I present a more ideal version of myself online”	1–5
Offline Social Competence	Adapted from the Interpersonal Communication Competence Scale (ICCS)	8	“I can clearly express my thoughts and opinions to others.”	1–5
Bonding Social Capital	Adapted from (Williams, 2006) and based on Woolcock's Social Capital framework	5	“I have close friends or family members whom I trust and can turn to for support.”	1–5
Bridging Social Capital	Adapted from (Williams, 2006) and based on Woolcock's Social Capital framework	5	“I interact with people from different backgrounds and gain access to new information and opportunities.”	1–5
Linking Social Capital	Adapted from (Williams, 2006) and based on Woolcock's Social Capital framework	5	“I can obtain resources and support through formal institutions such as universities and organizations.”	1–5
Loneliness	Adapted from the short UCLA Loneliness Scale	8	“I feel isolated from others.”	1–4

### 3.3 Model structure and settings

#### 3.3.1 PLS-SEM model estimation

This study employed SmartPLS 4.0 to estimate the proposed model using Partial Least Squares Structural Equation Modeling (PLS-SEM). PLS-SEM was selected instead of covariance-based structural equation modeling (CB-SEM) for several reasons.

First, the present study is primarily prediction-oriented and theory-extension oriented. The objective was to explain variance in endogenous constructs, particularly Loneliness, and to examine the relationships among emerging social interaction characteristics, Social Capital, and Loneliness in the context of artificial intelligence. PLS-SEM is particularly suitable for maximizing the explanatory power of endogenous variables and exploring predictive relationships.

Second, the proposed model contains multiple mediation pathways and relatively complex latent variable relationships, including the sequential associations among social interaction preferences, Social Presentation Preference, Social Capital, and Loneliness. Compared with CB-SEM, PLS-SEM is better suited for handling complex path models and mediation structures.

Third, PLS-SEM imposes fewer distributional assumptions and does not require strict multivariate normality, making it appropriate for behavioral and psychological survey data commonly encountered in social science research.

During data preprocessing, questionnaires containing substantial missing data were removed from the analysis. All first-order constructs in this study were specified as reflective constructs. The model was estimated using the path-weighting scheme in SmartPLS 4.0, with a maximum of 300 iterations and a stop criterion of 10^−7^. Statistical significance was assessed using a bootstrap procedure with 5,000 resamples.

Because all variables were collected through self-reported questionnaires, potential common method bias was also assessed using the full collinearity variance inflation factor (VIF) approach. The VIF values for all latent constructs ranged from 1.000 to 1.347, substantially below the recommended threshold of 3.3. Therefore, common method bias was unlikely to pose a serious threat to the validity of the findings.

#### 3.3.2 Research hypotheses

Based on the proposed theoretical framework and prior empirical findings, the present study advances the following hypotheses:

- H1: Social interaction preference positively predicts Social Presentation Preference.
- H2: Social Presentation Preference positively influences Social Capital, although the effect is relatively weak.
- H3: Offline Social Competence significantly and positively predicts Social Capital and represents the strongest predictive pathway.
- H4: Social Capital mediates the relationship between social interaction characteristics and Loneliness.
- H5: Social Capital negatively predicts Loneliness.
- H6: The indirect associations between social interaction characteristics and Loneliness through Social Capital differ in strength.

#### 3.3.3 Model specification

The research followed the standard procedures of Structural Equation Modeling (SEM) analysis.

During the initial model specification, several theoretically plausible direct paths—including the direct path from Social Interaction Preference to Social Capital—were estimated but did not reach statistical significance. Following the principle of model parsimony, these nonsignificant paths were removed from the final model because they lacked empirical support and their exclusion improved model interpretability without altering the theoretical framework. Model modification was conducted based on both statistical criteria and theoretical considerations. After eliminating nonsignificant paths, the revised model demonstrated improved model fit and explanatory power.

**Figure 1:**
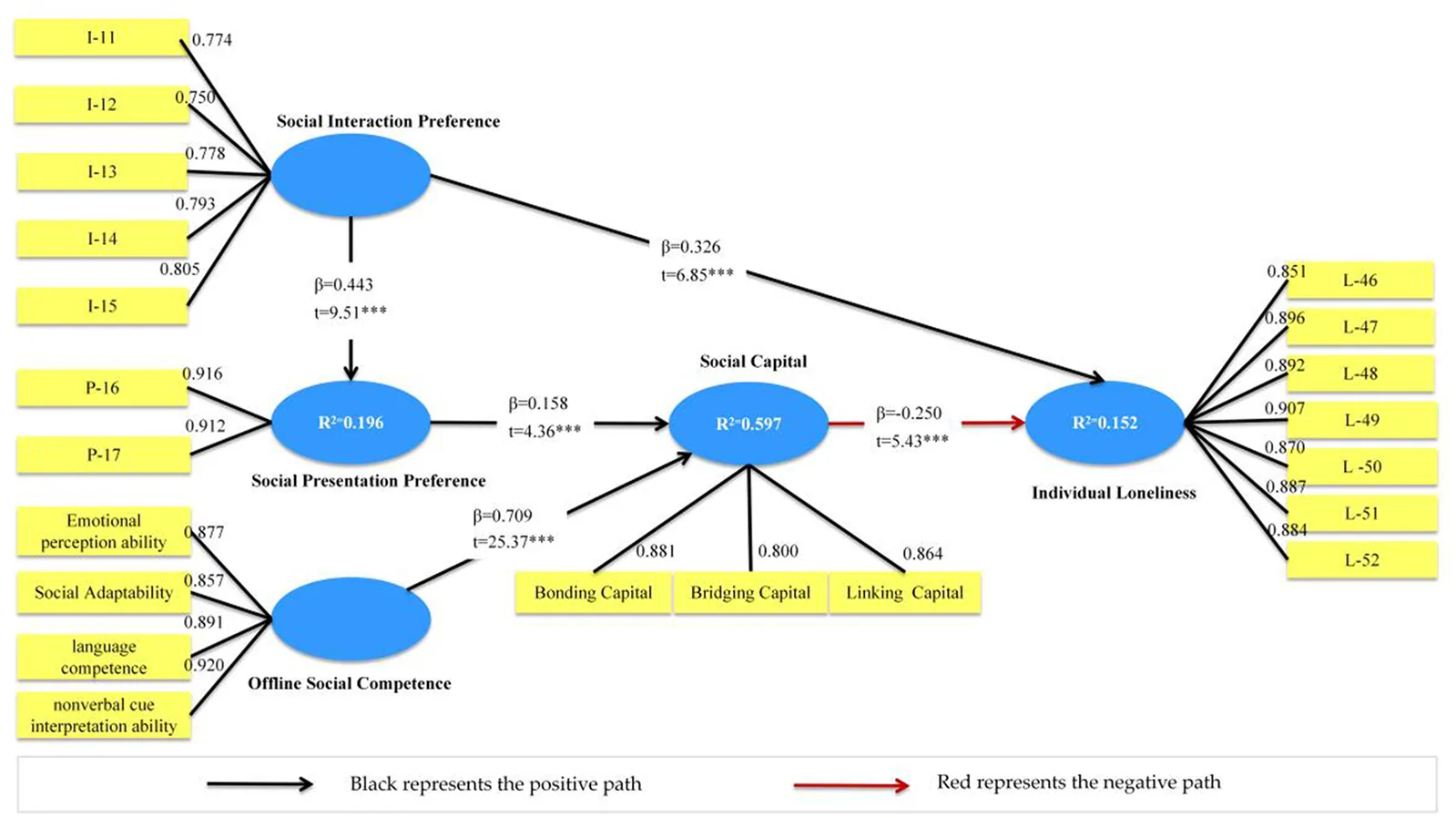
Final structural model.

The final structural model is presented in Figure 1.

### 3.4 Assessment of the measurement model

#### 3.4.1 Reliability and validity assessment

Reliability was primarily assessed using Cronbach's alpha and Composite Reliability (CR). The results indicate that the Cronbach's alpha values for all latent constructs ranged from 0.803 to 0.953, exceeding the acceptable threshold of 0.70. Similarly, the Composite Reliability values (CR; represented here by rho_c) ranged from 0.885 to 0.962, all substantially above the recommended standard of 0.70. These findings demonstrate that the measurement instruments employed in this study exhibit excellent internal consistency and high reliability.

Convergent validity reflects the degree of correlation among measurement items within the same latent construct. It was evaluated primarily through outer loadings and Average Variance Extracted (AVE). The AVE values for all constructs were well above the critical threshold of 0.50, indicating satisfactory convergent validity. This suggests that the measurement indicators effectively capture the conceptual content of their corresponding constructs. Detailed results are presented in Table 2.

**Table 2:** Reliability and validity assessment of the measurement model.

Latent construct	Cronbach's alpha	Composite reliability (rho_c)	AVE	Outer loadings
Social interaction preference	0.839	0.886	0.609	0.750–0.805
Social Presentation Preference	0.803	0.910	0.835	0.912–0.916
Offline Social Competence	0.909	0.936	0.786	0.857–0.920
Social Capital	0.805	0.885	0.721	0.800–0.881
Individual Loneliness	0.953	0.962	0.781	0.851–0.907

#### 3.4.2 Discriminant validity assessment (HTMT Values)

Discriminant validity was assessed to determine whether the latent constructs in the model were empirically distinct from one another. In this study, the Heterotrait–Monotrait Ratio (HTMT), which is considered one of the most rigorous approaches for evaluating discriminant validity, was employed for this purpose. The specific results are presented in Table 3.

**Table 3:** Discriminant validity assessment.

Constructs	Individual Loneliness	Social interaction preference	Social Presentation Preference	Offline Social Competence	Social Capital
Individual Loneliness	–				
Social interaction preference	0.335				
Social Presentation Preference	0.120	0.532			
Offline Social Competence	0.223	0.089	0.036		
Social Capital	0.244	0.148	0.468	0.884	–

The values represent inter-construct correlations used for discriminant validity assessment. Lower correlations among constructs indicate satisfactory discriminant validity and suggest that the latent variables capture conceptually distinct dimensions.

The HTMT results indicate that the values among the constructs predominantly ranged from 0.036 to 0.532, suggesting satisfactory discriminant validity for most construct pairs. The only relatively high HTMT value was observed between Offline Social Competence and Social Capital (HTMT = 0.884). Although this value slightly exceeded the conservative threshold of 0.85, it remained below the more liberal threshold of 0.90 recommended by Henseler et al. (2015). Therefore, discriminant validity was considered acceptable under the HTMT90 criterion.

Given the relatively high HTMT value and the strong structural relationship between Offline Social Competence and Social Capital, additional analyses were conducted to further evaluate potential discriminant validity concerns.

First, the Fornell–Larcker criterion provided additional support for discriminant validity. The square roots of the AVE values for Offline Social Competence (0.886) and Social Capital (0.849) both exceeded their inter-construct correlation (0.758), indicating satisfactory construct distinctiveness. The specific results are presented in Table 4.

**Table 4:** Fornell–Larcker criterion assessment.

Constructs	Individual Loneliness	Social interaction preference	Social Presentation Preference	Offline social competence	Social Capital
Individual Loneliness	**0.884**				
Social interaction preference	0.3	**0.78**			
Social Presentation Preference	0.105	0.443	**0.914**		
Offline social competence	−0.209	0.069	0.306	**0.886**	
Social capital	−0.215	0.106	0.376	0.758	**0.849**

Diagonal values (bold) represent the square roots of AVE. Discriminant validity is supported when each diagonal value exceeds all correlations in the corresponding row and column.

Second, cross-loading analysis demonstrated that all indicators loaded substantially higher on their intended constructs than on other constructs. Specifically, indicators of Offline Social Competence showed primary loadings ranging from 0.857 to 0.920, whereas their cross-loadings on Social Capital ranged from 0.645 to 0.694. Likewise, indicators of Social Capital showed primary loadings ranging from 0.800 to 0.881, whereas their cross-loadings on Offline Social Competence ranged from 0.627 to 0.659. These results further support the distinctiveness of the two constructs (Table 5).

**Table 5:** Additional discriminant validity evidence for Offline Social Competence and Social Capital.

Indicator	Primary loading	Highest cross-loading
Emotional perception	0.877	0.645
Social adaptability	0.857	0.694
Verbal expression ability	0.891	0.664
Nonverbal signal interpretation	0.920	0.681
Bonding Social Capital	0.800	0.659
Bridging Social Capital	0.881	0.641
Linking Social Capital	0.864	0.627

Finally, the two constructs are theoretically distinguishable. Offline Social Competence reflects individual-level social skills, including emotional perception, communication ability, social adaptation, and nonverbal signal interpretation. In contrast, Social Capital represents relationship-based resources and support obtained through social networks, encompassing bonding, bridging, and linking Social Capital. Therefore, the former reflects an individual capability, whereas the latter represents a social resource outcome.

Taken together, the HTMT assessment, Fornell–Larcker criterion, cross-loading evidence, and theoretical distinctions consistently support the discriminant validity of the measurement model. Although Offline Social Competence and Social Capital were strongly associated, they remain conceptually and empirically distinct constructs with independent theoretical significance and were therefore retained as separate constructs in the structural model.

#### 3.4.3 Collinearity assessment

The variance inflation factor (VIF) values for the internal model ranged from 1.000 to 1.109. All VIF values were significantly below the collinearity threshold of 3.3 (or 5.0), indicating no collinearity among the independent variables and thus no negative impact on the accuracy of the path coefficients.

#### 3.4.4 Structural model assessment

Following the evaluation of the measurement model, the structural model was assessed by examining model fit, explanatory power, effect sizes, and predictive relevance.

The standardized root mean square residual (SRMR) value was 0.077, which is below the recommended threshold of 0.08, indicating an acceptable model fit.

Regarding explanatory power, the *R*^2^ values were 0.196 for Social Presentation Preference, 0.597 for Social Capital, and 0.152 for Loneliness. These results suggest that the model provides substantial explanatory power for Social Capital and moderate explanatory power for Social Presentation Preference and Loneliness.

The corresponding adjusted *R*^2^ values were 0.194, 0.595, and 0.148, respectively (Table 6).

**Table 6:** Coefficient of determination (*R*^2^).

Endogenous construct	*R^2^*	Adjusted *R^2^*
Social Presentation Preference	0.196	0.194
Social Capital	0.597	0.595
Loneliness	0.152	0.148

Effect size analysis revealed that Offline Social Competence exerted a particularly strong effect on Social Capital (f^2^ = 1.132), representing the largest effect in the model. Social Interaction Preference demonstrated a moderate effect on Social Presentation Preference (f^2^ = 0.244), whereas the remaining paths showed small-to-moderate effects (Table 7).

**Table 7:** Effect size assessment (f^2^).

Path	f^2^
Social Interaction Preference → Social Presentation Preference	0.244
Social Presentation Preference → Social Capital	0.056
Offline Social Competence → Social Capital	1.132
Social Capital → Loneliness	0.073
Social Interaction Preference → Loneliness	0.124

According to (Cohen, 1988), f^2^ values of 0.02, 0.15, and 0.35 indicate small, medium, and large effects, respectively.

To further assess predictive relevance, the PLSpredict procedure was conducted. The Q^2^predict values for Loneliness (0.131), Social Presentation Preference (0.189), and Social Capital (0.571) were all above zero, indicating satisfactory predictive relevance. Among these constructs, Social Capital demonstrated strong predictive performance, whereas Loneliness and Social Presentation Preference exhibited moderate predictive relevance (Table 8).

**Table 8:** Predictive relevance assessment (Q^2^predict).

Construct	Q^2^predict	RMSE	MAE
Individual Loneliness	**0.131**	0.936	0.766
Social Presentation Preference	0.189	0.906	0.692
Social Capital	0.571	0.659	0.505

Q^2^predict values greater than zero indicate predictive relevance. According to (Hair et al., 2022), values of approximately 0.02, 0.15, and 0.35 indicate small, medium, and large predictive relevance, respectively.

Overall, the proposed model demonstrated satisfactory model fit, explanatory power, and predictive capability.

## 4 Data analysis and discussion

### 4.1 Detailed results of path coefficients

The *t*-values for all pathways were greater than 1.96 (*p* < 0.05), indicating that all relationships passed the significance test and demonstrate statistically significant correlations among the variables.

Path coefficients (β) are core parameters in Structural Equation Modeling (SEM), representing the strength and direction of the direct relationships between variables. The magnitude and sign of the coefficients indicate both the intensity and directionality of these relationships. Path coefficients generally range from −1 to +1. Values close to 0 indicate little or no linear relationship, whereas values approaching ±1 indicate a strong linear relationship. According to conventional criteria, a coefficient around 0.10 represents a small effect, approximately 0.30 indicates a medium effect, and 0.50 or above reflects a large effect.

Specifically, the path from social interaction preference to Social Presentation Preference showed a relatively strong positive effect (β= 0.443, *t* = 9.505, *p* < 0.001). Social Presentation Preference positively predicted Social Capital, although the effect size was relatively weak (β = 0.158, *t* = 4.359, *p* < 0.001). Offline Social Competence emerged as the strongest predictor of Social Capital (β= 0.709, *t* = 25.369, *p* < 0.001), indicating a very strong positive effect. In addition, social interaction preference directly and positively predicted individual Loneliness (β = 0.326, *t* = 6.851, *p* < 0.001), while Social Capital negatively predicted Loneliness (β = -0.250, *t* = 5.431, *p* < 0.001), suggesting that higher levels of Social Capital significantly alleviated Loneliness (Table 9).

**Table 9:** Results of structural path coefficients.

Path	Path coefficient (β)	*t*-value	*p*-value	Significance	Direction	Effect size
Social Interaction Preference → Individual Loneliness	0.326	6.851	< 0.001	^***^	Positive	Medium
Social Interaction Preference → Social Presentation Preference	0.443	9.505	< 0.001	^***^	Positive	Relatively strong
Social Presentation Preference → Social Capital	0.158	4.359	< 0.001	^***^	Positive	Weak
Offline Social Competence → Social Capital	0.709	25.369	< 0.001	^***^	Positive	Very strong
Social Capital → W Individual Loneliness	−0.250	5.431	< 0.001	^***^	Negative	Medium

^***^p < 0.001.

**Figure 2:**
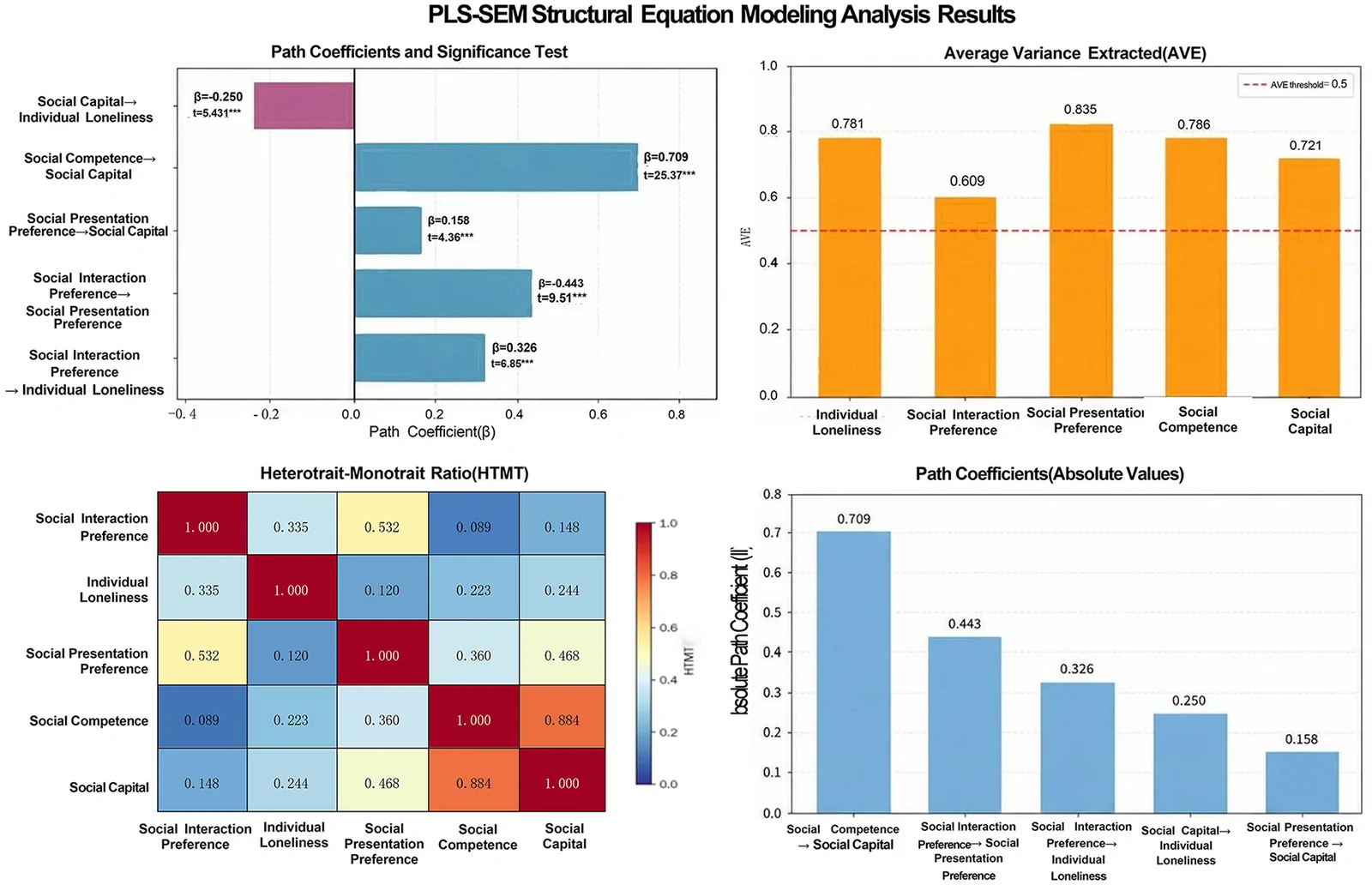
PLS-SEM structural equation modeling analysis results.

The specific results of PLS-SEM structural equation modeling analysis are shown in Figure 2.

### 4.2 Direct effects

The structural model revealed several significant direct associations among the core constructs.

Social Interaction Preference was positively associated with both Social Presentation Preference (β = 0.443, *p* < 0.001) and Individual Loneliness (β = 0.326, *p* < 0.001). These findings suggest that students who rely more heavily on online social interaction tend to engage more frequently in self-presentation behaviors while also reporting higher levels of Loneliness.

Social Presentation Preference was positively associated with Social Capital (β = 0.158, *p* < 0.001), although the magnitude of this relationship was relatively modest. This result indicates that online self-presentation may contribute to social resource accumulation, but its association with Social Capital remains limited.

In contrast, Offline Social Competence demonstrated the strongest positive association with Social Capital in the model (β = 0.709, *p* < 0.001). This finding highlights the close relationship between face-to-face social abilities and the accumulation of social resources.

Finally, Social Capital was negatively associated with Individual Loneliness (β = −0.250, *p* < 0.001), suggesting that students with greater access to social resources and support tend to report lower levels of Loneliness.

Taken together, the direct-effect results indicate that online and offline social interaction characteristics are associated with Loneliness through different pathways and exhibit substantially different relationships with Social Capital.

### 4.3 Mediation effect analysis

Further analysis revealed significant differences across the mediating pathways. To further examine the indirect relationships proposed in the model, mediation effects were assessed using a bootstrap procedure with 5,000 resamples. Indirect effects, *t*-values, *p*-values, and 95% bootstrap confidence intervals were estimated.

The results indicated that Social Interaction Preference exerted a significant indirect association with Loneliness through the sequential pathway of Social Presentation Preference and Social Capital [Indirect Effect = −0.017, *t* = 3.119, *p* < 0.001, 95% CI (−0.028, −0.009)]. Because the confidence interval did not include zero, the chain mediation effect was considered statistically significant.

In addition, Offline Social Competence demonstrated a significant indirect association with Loneliness through Social Capital [Indirect Effect = −0.177, *t* = 5.303, *p* < 0.001, 95% CI (−0.243, −0.113)]. Likewise, Social Presentation Preference was indirectly associated with Loneliness through Social Capital [Indirect Effect = −0.040, *t* = 3.298, *p* < 0.001, 95% CI (−0.061, −0.021)] Furthermore, Social Interaction Preference showed a significant indirect association with Social Capital through Social Presentation Preference [Indirect Effect = 0.070, *t* = 3.960, *p* < 0.001, 95% CI (0.043, 0.101)].

Taken together, these findings support the mediating role of Social Capital within the proposed model and reveal notable differences in the strength of the indirect pathways.

First, although Social Interaction Preference did not exhibit a significant direct association with Social Capital, it was indirectly associated with Social Capital through Social Presentation Preference. This finding suggests that online self-presentation and identity construction may contribute to the accumulation of certain social resources, although the strength of this pathway was relatively modest.

Second, Social Presentation Preference was indirectly associated with lower levels of Loneliness through Social Capital. This result suggests that online self-presentation and identity construction may be associated with reduced Loneliness by facilitating the accumulation of social resources, although the magnitude of the effect remained relatively limited.

Third, the sequential pathway linking Social Interaction Preference, Social Presentation Preference, Social Capital, and Loneliness was statistically significant but comparatively small in magnitude. This finding indicates that the compensatory association of online social interaction with Loneliness through Social Capital accumulation was relatively limited.

Finally, the indirect pathway linking Offline Social Competence, Social Capital, and Loneliness was substantially stronger than the online-mediated pathways. Among all indirect effects examined, this pathway demonstrated the largest effect size, suggesting that Offline Social Competence was more strongly associated with Social Capital accumulation and lower levels of Loneliness.

### 4.4 Differential indirect associations

Overall, the findings reveal a clear pattern of differential indirect assocations. The indirect pathway linking Offline Social Competence, Social Capital, and Loneliness was substantially stronger than the indirect pathway involving online self-presentation and Social Capital.

Meanwhile, this study identified a typical inconsistent mediation mechanism. Although social interaction preference could indirectly reduce Loneliness through the accumulation of Social Capital, its direct positive effect on Loneliness was considerably stronger. These findings suggest that the indirect association between online social interaction preferences and Loneliness through Social Capital was comparatively small relative to the direct positive association observed in the model.

## 5 Conclusion

Grounded in Social Capital theory and employing Partial Least Squares Structural Equation Modeling (PLS-SEM), this study examined the differentiated mechanisms linking university students' social interaction characteristics and Loneliness in the age of artificial intelligence. The major findings are summarized as follows.

### 5.1 The dual mechanism of online social interaction preference on loneliness

By revealing the dual-path mechanism of digital social interaction, this study provides a more nuanced explanation of the relationship between university students' digital social engagement and Loneliness. On the one hand, individuals may accumulate a certain degree of Social Capital through online interaction and identity construction, thereby alleviating Loneliness to some extent. However, this indirect effect was extremely weak, indicating that the psychological compensation generated by digital social interaction is limited. On the other hand, excessive reliance on online social interaction may weaken authentic interpersonal connections and significantly increase Loneliness, with a comparatively stronger direct positive effect.

This finding integrates the “social compensation theory” and the “social displacement theory,” suggesting that the two perspectives are not mutually exclusive but coexist with different levels of influence. Ultimately, the displacement effect appears to dominate, resulting in increased Loneliness.

### 5.2 Social capital from different sources exhibits differentiated mediating effects

From the perspective of Social Capital theory, the impact of social interaction on Loneliness depends not only on the quantity of social connections but also on the pathways through which Social Capital is accumulated. The study identified significant differentiated mediating effects, demonstrating that Social Capital is not a homogeneous resource.

The pathway from Offline Social Competence to Social Capital was significantly stronger than the pathway from Online Interaction Preferences and Image Presentation to Social Capital. This finding may indicate that Offline Social Competence is more strongly associated with Social Capital accumulation, it demonstrates strong predictive power and a substantial buffering effect against Loneliness.

In contrast, the association between online self-presentation and Social Capital was comparatively weaker. Although it may help expand social networks, its association with Social Capital accumulation was comparatively limited, resulting in only a modest indirect association with Loneliness, exerting only a very limited effect on alleviating Loneliness.

These findings extend the application of Social Capital theory within AI-mediated contexts by emphasizing that social resources differ not only in their quantity but also in their pathways of accumulation and functional associations with Loneliness.—differences that become especially pronounced in the age of artificial intelligence. This study therefore offers a new explanatory pathway for understanding the paradox of “increased connectivity yet intensified Loneliness” in the digital era.

### 5.3 The dominant role of offline social competence

The empirical results indicate that Offline Social Competence is the strongest predictor of Social Capital within the proposed model. Compared with pathways relying on online identity construction, Offline Social Competence demonstrated substantially greater explanatory power and played a more fundamental and critical role in the generation of social resources. Furthermore, it significantly reduced Loneliness through the accumulation of Social Capital.

These findings suggest that real-world interaction abilities remain a key influencing factors for individual Social Capital and psychological wellbeing. The results challenge the implicit assumption that “digital natives” primarily depend on online interaction and instead highlight the irreplaceable role of embodied interaction and offline communication skills in establishing high-quality social relationships.

Particularly for university students undergoing a transitional stage of socialization, insufficient Offline Social Competence may reduce opportunities for participation in real-world social activities, hinder the accumulation of Social Capital, and ultimately intensify Loneliness.

### 5.4 Inconsistent mediation and the limits of digital compensation

The inconsistent mediation mechanism identified in this study further reveals the limitations of digital social compensation. Although online social interaction preference and online social presentation can indirectly reduce Loneliness through Social Capital, their direct positive effects on Loneliness are substantially stronger, indicating structural limitations in the compensatory effects of digital social interaction.

In other words, while digital interaction may provide a certain sense of connectedness, such connections often lack emotional depth and authenticity, making them insufficient for generating enduring psychological support. Consequently, the “micro-level compensation” produced by online interaction cannot offset the “macro-level deficit” caused by the reduction of offline interaction. From a theoretical perspective, the findings may imply that digitally mediated interaction and face-to-face interaction serve different social functions. Technological development alone cannot replace human beings' fundamental need for authentic social relationships, although further longitudinal research is needed to verify these interpretations.

### 5.5 Practical implications

This study provides important implications for understanding social development in the age of artificial intelligence. As digital technologies continue to reshape patterns of interpersonal interaction, it becomes increasingly important to achieve a complementary balance between online social engagement and Offline Social Competence in order to facilitate the effective accumulation of Social Capital.

For university students, who are at a critical transitional stage of socialization, excessive reliance on digital interaction may weaken the development of Offline Social Competence and limit their ability to establish stable and supportive social networks. Therefore, cultivating Offline Social Competence should be regarded as an important pathway for promoting psychological wellbeing.

From the perspective of higher education practice, several implications emerge. First, educational interventions should return to a focus on “competence development” rather than merely “platform adaptation,” emphasizing the cultivation of students' Offline Social Competence through diverse campus activities that enhance embodied social participation. Second, mental health interventions should pay greater attention to improving real-world interpersonal communication abilities rather than relying solely on digital communication tools. Third, universities should pay particular attention to first-year students during their adjustment period, guiding them to avoid excessive dependence on online interaction while encouraging the development of real-world social networks.

## 6 Limitations and future research

Despite its contributions, this study has several limitations. First, the cross-sectional research design limits the reliability of causal inference. Second, the observational indicators of the independent variables could be further refined, and broader sample expansion may improve measurement precision.

Future research could incorporate longitudinal designs to further examine how AI-specific interaction patterns shape social relationships and psychological outcomes over time. Because the present study employed a cross-sectional design, the observed relationships should be interpreted as associations rather than causal effects. Future longitudinal and experimental studies are needed to examine the temporal dynamics and causal mechanisms linking social interaction, Social Capital, and Loneliness.

## Data Availability

The raw data supporting the conclusions of this article will be made available by the authors, without undue reservation.
